# Anti-programmed cell death protein 1 (anti-PD1) immunotherapy induced autoimmune polyendocrine syndrome type II (APS-2): a case report and review of the literature

**DOI:** 10.1186/s40425-019-0713-y

**Published:** 2019-09-05

**Authors:** Ashray Gunjur, Oliver Klein, Damien Kee, Jonathan Cebon

**Affiliations:** 1 Department of Medical Oncology, Austin Health, Melbourne, Australia; 2 Department of Medicine, University of Melbourne, Melbourne, Australia; 3 Olivia Newton-John Cancer Research Institute, School of Cancer Medicine, La Trobe University, Melbourne, Australia

**Keywords:** Immune checkpoint inhibitor, PD1 inhibitor, Pembrolizumab, Poly-endocrinopathy, Diabetes mellitus, Hypoadrenalism, Hypothyroidism

## Abstract

**Background:**

Autoimmune polyendocrine syndrome type II (APS-2) is a rare constellation of autoimmune hypoadrenalism, thyroid dysfunction and/or type 1 diabetes (T1DM), usually occurring in the 3rd or 4th decades and associated with a human leukocyte antigen (HLA) DR3 or DR4 serotype. We detail the first report of an elderly woman developing the full triad of APS-2 shortly after commencing anti-programmed cell death protein 1 (anti-PD1) immune checkpoint inhibition for unresectable melanoma and review the literature for similar presentations secondary to anti-PD1 axis therapy.

**Case:**

A 78-year-old female with advanced unresectable BRAF wild-type melanoma was treated with pembrolizumab (2 mg/kg 3-weekly). Three weeks following the first dose she developed fulminant autoimmune diabetes, with an initially low C-peptide denoting rapid destruction of ß-islet cells. Following stabilisation of her diabetes, two further doses of pembrolizumab was administered. She then represented with symptomatic hypoadrenalism and hypothyroidism, consistent with APS-2. Her HLA class II genotype was HLA-DRB1*04.16 (DR4 serotype), a recognised association with this syndrome. Her melanoma responded rapidly to anti-PD1 therapy, and a complete response (CR) was attained after four doses of pembrolizumab. Treatment was discontinued and her CR is ongoing.

**Conclusion:**

This is the first report of the full triad of APS-2 developing in a genetically susceptible individual at the age of 78 after treatment with an anti-PD1 agent. Although scarcely reported, a literature review of similar reports seems to indicate a predilection for this syndrome in patients with HLA-DR4 serotypes. HLA Class II typing is not routinely recommended, but may provide useful predictive information for patients at risk of poly-endocrinopathy even in patients without a relevant personal or family history. Additional studies are required to determine if such testing would be useful and/or cost effective.

**Electronic supplementary material:**

The online version of this article (10.1186/s40425-019-0713-y) contains supplementary material, which is available to authorized users.

## Background

Immune checkpoint inhibitors targeting the programmed cell death protein 1 (PD1) or its ligand (PD-L1) have revolutionised the treatment of many malignancies, particularly melanoma, non-small cell lung cancer (NSCLC) and renal cell carcinoma (RCC), however they are not without side effects. These are chiefly inflammatory, often termed “immune related adverse events” (irAEs) and can affect virtually any organ system, including endocrine glands.

Of the anti-PD1-related endocrinopathies thyroiditis is most frequently seen, causing hypothyroidism in approximately 6.0% and hyperthyroidism in 2.8% of anti-PD1/PD-L1 treated patients (though these data may not capture the proportion of patients transitioning from initial hyperthyroidism to hypothyroidism, which often occurs). Autoimmune diabetes mellitus (DM) or adrenal insufficiency are observed much less frequently; with an all-grade incidence of only 0.4 and 0.69% respectively [1].

In 1926, Schmidt described two cases of primary adrenal insufficiency coinciding with the occurrence of auto-immune thyroiditis, thereafter eponymously termed “Schmidt syndrome” [2]. Contemporary groups refer to the syndrome as “polyglandular autoimmune syndrome type II” or “autoimmune polyendocrine syndrome type II” (APS-2) with variable definitions, some defining it to occur if any two of three of type 1 diabetes mellitus (T1DM), autoimmune thyroiditis and primary hypoadrenalism occur, while others specifying hypoadrenalism must occur, with at least one of the other two conditions. In either case, APS-2 has a predilection to women; usually develops in the 3rd to 4th decade of life; and is thought to be polygenetic, with mutations in cytotoxic T lymphocyte-associated antigen 4 (CTLA-4, another immune checkpoint inhibitor (ICI) target) and human leukocyte antigen (HLA) DR3 and DR4 serotypes being known associations [3, 4].

Here we report a case of an elderly female who sequentially developed all three features of APS-2 shortly after starting treatment with the anti-PD1 antibody pembrolizumab, with testing confirming a HLA-DRB1*04 genotype (DR4 serotype). Our case description is supplemented by a literature review of PD1/PD-L1 inhibitor associated APS-2.

## Case presentation

**Fig. 1:**
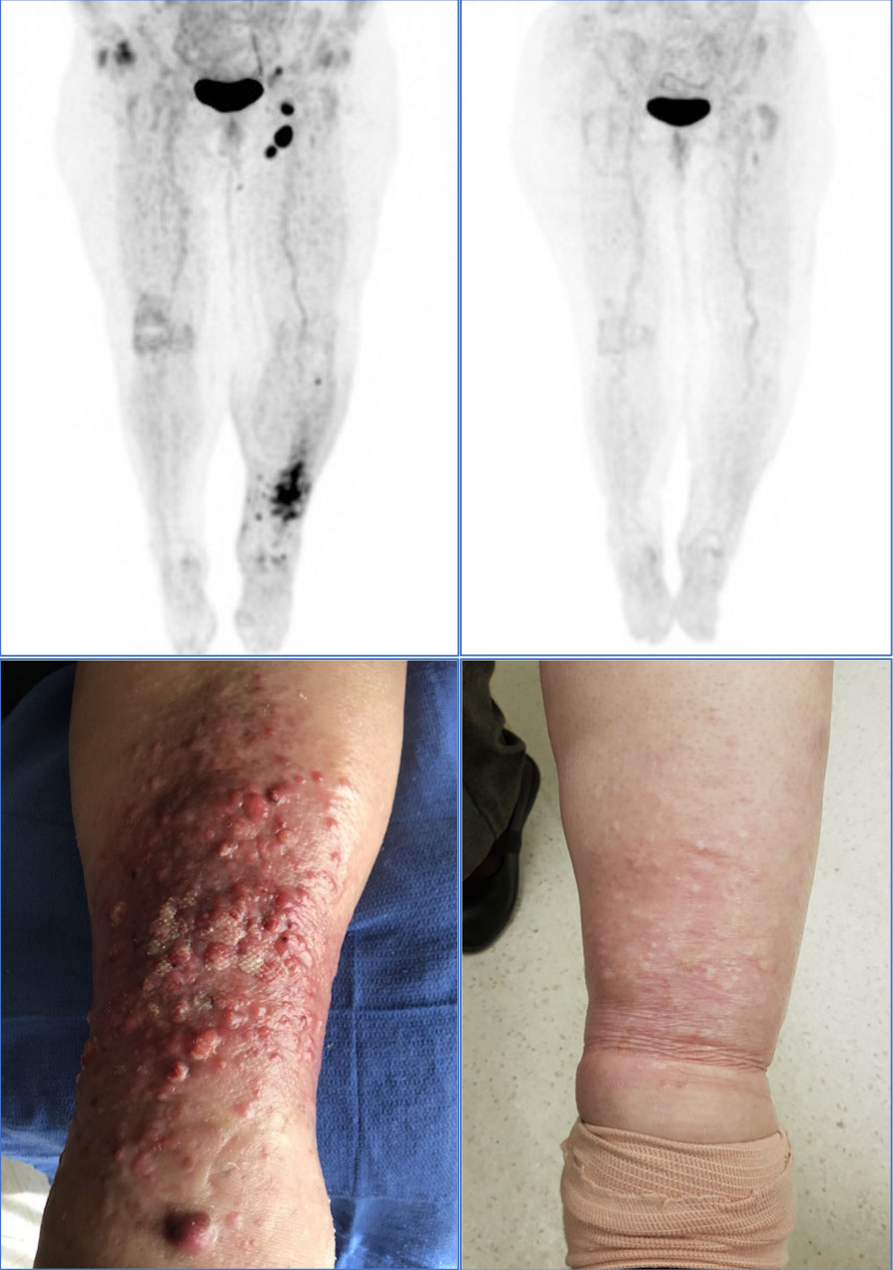
FDG-PET and Clinical Response. FDG-PET maximum intensity projection in July 2018, prior to pembrolizumab treatment (top left panel) and in April 2019, over 6 months after her fourth and final pembrolizumab treatment (top right), with corresponding clinical photography of the patient's left shin from these time points (bottom left and bottom right respectively). In 2018, lesions over left shin were erythematous non-tender papules and nodules, with biopsy confirming metastatic melanoma

A 77-year-old woman of Italian ancestry initially presented with an ulcerated acral melanoma (left heel) in mid-2017 (Breslow depth 2.1 mm, BRAF wild-type). Sentinel lymph node biopsy was negative. She presented 12 months later (mid-2018) with left lower limb lymphoedema and multiple new subcutaneous nodules over that shin. A Fluorodeoxy-glucose (FDG) Positron Emission Tomography (PET) scan demonstrated FDG-avid regional recurrence associated with bulky ipsilateral inguinal and external iliac lymph nodes (Fig. 1). Biopsy of a subcutaneous nodule confirmed in-transit metastatic melanoma (confirming T3bN3M0 disease, stage IIIC by the American Joint Committee on Cancer (AJCC) 8th edition). Co-morbidities included asthma, treated with inhaled corticosteroids and salbutamol, paroxysmal atrial fibrillation, gastro-esophageal reflux disease and hypertension. There was no past or family history of autoimmune or endocrine disease.

Her in-transit and nodal disease was considered unresectable and she was treated with single-agent PD1-inhibitor pembrolizumab (2 mg/kg every 3 weeks), receiving her first dose in July 2018. Baseline blood tests revealed a random glucose of 10.5 mmol/L (normal range: 4.4–8.9 mmol/L) (189 mg/dL), sodium of 133 mmol/L (normal range: 135–145 mmol/L) and thyroid stimulating hormone (TSH) 2.34 mU/L (normal range: 0.27–4.20 mU/L).

Three weeks later the patient reported approximately 7 days of worsening malaise, nausea and polyuria. Blood tests revealed a low serum sodium of 120 mmol/L, osmolality of 306 mmol/kg (normal range: 275–29.5 mmol/kg), glucose of 44.0 mmol/L (792 mg/dL), ketones 1.8 mmol/L (normal range: 0–0.6 mmol/L) and bicarbonate 18 mmol/L (normal range: 22–30 mmol/L). The calculated anion gap was 19.8, indicating a high-anion gap acidosis. These results were in keeping with fulminant DM with associated pseudohyponatremia.

Emergency management included an insulin infusion and intravenous rehydration, resulting in normalisation of blood glucose and resolution of ketosis. Additional blood tests revealed a mildly elevated HbA1c of 6.9% (normal range: < 6.5%), a low C-peptide of 0.22 nmol/L (normal range: 0.33–1.47 nmol/L) and strongly positive auto-antibodies of anti-Glutamic Acid Decarboxylase (anti-GAD) > 2000.0 U/ml (normal range: < 5.0 U/mL) and anti-islet antibody 2 (anti-IA2) 871 U/mL (normal range: < 15.0 U/mL), consistent with T1DM. She was commenced on 40 units of insulin degludec/insulin aspart 70/30 (combination ultra-long and short acting insulin) daily and received her second dose of pembrolizumab on her day of discharge. Three weeks later (early September 2018) the patient had clinically stabilised and had normal thyroid studies (TSH 2.52 mU/L, free T4 17.28 mU/L), 8 am cortisol (347 nmol/L, normal range: 140–490 nmol/L) and low-normal sodium (133 mmol/L). She received her third dose of pembrolizumab.

**Fig. 2:**
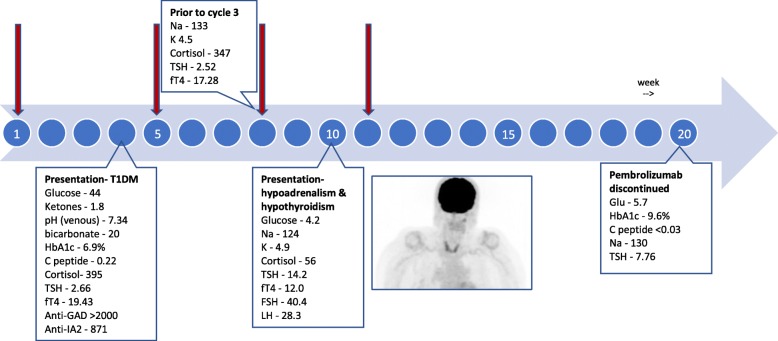
Time Course. *Red arrows indicate pembrolizumab doses, with blue dots indicating passing weeks. FDG-PET maximum intensity projection image was taken at week 10, demonstrating thyroiditis. Laboratory normal reference ranges & units: Random glucose: 4.4–8.9 mmol/L (79–160 mg/dL). Ketones: 0–0.6 mmol/L. Sodium (Na): 135–145 mmol/L. Potassium (K): 3.5–5.2 mmol/L. Bicarbonate: 22–30 mmol/L. Cortisol: 140–490 nmol/L. TSH: 0.27–4.20 mU/L. fT4: 12.0–22.0 mU/L. follicular stimulating hormone (FSH): 25.8–134.8 IU/L (post-menopause). luteinizing hormone (LH): 14.2–52.3 IU/L (post-menopause). HbA1c: < 6.5%. C peptide: 0.33–1.47 nmol/L*

Prior to cycle 4 of pembrolizumab (in late-September 2018) the patient re-presented to the emergency department, now with significant fatigue, functional decline and nausea. The patient’s blood pressure was 107/68 mmHg (with previous systolic measurements never below 120 mmHg), heart rate 59/min, respiratory rate 20/min and temperature 35.0 °C (95.0 °F). Serum sodium was once again noted to be low (124 mmol/L), this time with a low-normal serum glucose of 4.2 mmol/L (75.6 mg/dL), thus hyperglycaemia not artifactually contributing to the hyponatraemia. Her potassium was high-normal at 4.9 mmol/L (normal range 3.5–5.2 mmol/L), TSH elevated at 14.2 mU/L (normal range: 0.27–4.20 mU/L), free T4 borderline-low at 12.0 mU/L (normal range: 12.0–22.0 mU/L) and other anterior pituitary hormones within normal limits (Fig. 2). Hypocortisolaemia was confirmed (56 nmol/L) and the patient was commenced on exogenous corticosteroids (initially intravenous hydrocortisone, then transitioned to oral cortisone 20 mg mane, 10 mg nocte) with rapid clinical improvement. Hypoadrenalism was diagnosed by a short cosyntropin (a synthetic corticotropic agent) test: 250mcg cosyntropin was administered intravenously and failed to elicit an appropriate cortisol increment after 60 min.

A restaging FDG-PET/CT at this time point demonstrated a near complete response (CR) of the previously FDG-avid in-transit and nodal metastases; no morphological change or metastases to the adrenals; and incidental diffuse FDG uptake in the thyroid consistent with thyroiditis (Fig. 2). Primary hypothyroidism was diagnosed on the basis of a high TSH and borderline low free T4 and replacement therapy with thyroxine was commenced at 50mcg per day. Once stabilised and on the day of her discharge she received her fourth and final dose of pembrolizumab.

Three weeks following discharge (in mid-October 2018) repeat thyroid function tests demonstrated persistent hypothyroidism (TSH 40.4 mU/L, free T4 11.68 mU/L) and the patient’s thyroxine replacement was increased to 75mcg per day. A normal MRI of brain, high TSH and the absence of headache helped exclude hypopituitarism as a contributing cause of endocrine dysfunction in this patient. Given the patient’s frailty and difficulty attending for infusions, she opted to discontinue further therapy with pembrolizumab.

At the time of writing, almost 1 year after her last dose of pembrolizumab, the patient remains in clinical and radiological remission and is now stable on (likely lifelong) insulin, cortisone and thyroxine replacement. Her HbA1c is 9.6% (indicating worsening diabetic control), while her C-peptide is no longer detectable (consistent with destruction of pancreatic ß-islet glands and no endogenous production of insulin). Patient HLA class II allele typing demonstrated HLA-DRB1*04.16, DQB1*02.05 and DQA1*01.03 allotypes.

## Discussion and conclusions

To our best knowledge we report the first case of the full triad of APS-2: diabetes mellitus, primary hypoadrenalism and hypothyroidism, following monotherapy with a PD1 inhibitor.

Immune-related DM as a consequence of anti-PD1 axis therapy is an uncommon but documented phenomenon, reported in approximately 0.4% receiving anti-PD1/PD-L1 monotherapy [1]. Our patient’s short (1 week) prodrome of symptoms, initially low C-peptide (0.22 nmol/L) and only modestly elevated initial HbA1c (6.9%) point to acute, rapid destruction of ß-islets in the pancreas. This is consistent with the case details from the largest published series of anti-PD1 therapy induced DM (27 patients), for whom 88% had low or undetectable C-peptide at diagnosis. In that series, DM developed a median of 20 weeks after starting anti-PD1 therapy (with a wide range 1–228 weeks, indicating a stochastic nature to this event) and interestingly, only 40% (10 of 25) had one positive T1DM auto-antibody. Those who were ‘seropositive’ for T1DM (as our patient was) tended to develop it sooner after starting anti-PD1 therapy (at a median of 2.5 versus 13 cycles) [5].

Spontaneous APS-2 is rare, with an estimated prevalence of 1.4–2.0 per 100,000 Caucasians (and a 3:1 predilection for women) [3]. It is thought to be polygenetic, with a large study of 98 German patients with spontaneous APS-2 showing a similar pattern of HLA class II alleles to people with spontaneous T1DM (with significantly higher representation of HLA-DR3 (28.6%) and DR4 (35.2%) serotypes than matched health controls (10.6 and 12.6% respectively) [6]. To review all currently published cases of APS-2 triggered by anti-PD1 therapy we conducted a comprehensive structured MEDLINE® search using a combination of Medical Subject Headings (MeSH) terms and keywords (Additional file 1). A secondary search of the bibliographies of all included manuscripts was also undertaken, in total finding 13 relevant cases (Table 1). Of these, only two cases experienced primary hypoadrenalism and none reported all three features of APS-2, making this report unique.

**Table 1:** Summary of Case Reports of anti-PD1/PD-L1 induced APS-2 (at least two components)

Author (year published)	Therapy	Age (years)	Country	Disease	Timing^a^ (weeks)	Conditions	auto-Ab found	HLA type	Response
Hakami [7] (2019)	pembrolizumab	52	Ireland	melanoma	21	hypothyroidism T1DM		NR	NR
Lanzolla [8] (2019)	atezolizumab	60	Italy	NSCLC	6	T1DM hypoadrenalism	anti-21-OH	DRB1*04 DQB1*03	PD
Sakurai [9] (2018)	nivolumab	68	Japan	RCC	14	hypothyroidism^b^ T1DM	anti-TPO Anti-Tg	DRB1*09:01 DQB1*03:03	NR
Gauci [10] (2017)	nivolumab	73	France	melanoma	6	hyperthyroidism^b^ T1DM	anti-GAD anti-IA2 anti-ZnT8	NR	PR
Paepegaey [11] (2017)	pembrolizumab	55	France	melanoma	12	hypothyroidism hypoadrenalism	anti-21-OH	NR	NR
Li [12] (2017)	nivolumab	63	USA	NSCLC	4	T1DM hypothyroidism	anti-GAD anti-TPO	NR	PD
Alhusseini [13] (2017)	pembrolizumab + ipilimumab	65	USA	NSCLC	3	T1DM hyperthyroidism	anti-GAD anti-IA anti-insulin anti-TPO	NR	PR
Lowe [14] (2016)	nivolumab + ipilimumab	54	USA	melanoma	2	hyperthyroidism T1DM	anti-GAD	DQB1*0602	CR
Kong [15] (2016)	pembrolizumab	68	Korea	NSCLC	21	T1DM hyperthyroidism	Nil	DRB1*09:01 DQB1*03:03	PR
Hansen [16] (2016)	pembrolizumab	58	USA	melanoma	NR	hypothyroidism T1DM	anti-GAD	NR	PR
Mellati [17] (2015)	NR	66		SCC jaw	7	T1DM hypothyroidism	anti-TPO anti-GAD	DR3-DQ2 DR4-DQ8	NR
Hughes [18] (2015)	nivolumab + ipilimumab	55	USA	melanoma	20	hypothyroidism^b^ T1DM		A2.1 DR4	NR
Hughes (2015)	pembrolizumab	64	USA	melanoma	< 4	hypothyroidism^b^ T1DM		DR4	NR

*Abbreviations*: *auto-Ab* auto-antibody, *HLA* Human leukocyte antigen, *T1DM* Type 1 diabetes mellitus, *NR* Not reported, *PD* Progressive disease, *21-OH* 21-hydroxylase, *anti-TPO* Anti-thyroid peroxidase, *anti-Tg* anti-thyroglobulin, *anti-GAD* anti-glutamic acid decarboxylase, *anti-IA2* anti-islet antibody 2, *anti-ZnT8* anti-zinc transporter 8, *PR* partial response, *CR* complete response

^a^timing denotes weeks after start of anti-PD1 therapy to onset of APS-2

^b^auto-immune condition preceded treatment with anti-PD1 axis therapy

Our patient was 78 years old when she developed APS-2, being the oldest in the identified cases (ranging from 52 to 73 years old). This is striking when considering the average age of developing spontaneous APS-2 is thought to be 30–40 years old. Including ours, 8 of 14 case reports performed some form of HLA typing, of whom 5 (63%) were HLA-DR4. This appears somewhat higher than the rate of HLA-DR4 in patients with spontaneous APS-2 (35.2%), but is similar to that for anti-PD1 therapy induced DM (76% in the previously cited case series) [5]. The association of HLA class and susceptibility to irAEs is not well studied, however the well-established relationship between HLA class and spontaneous autoimmunity suggest that this is likely. If so, HLA class II haplotype may serve as a useful biomarker for predicting risk of irAEs – endocrinological and potentially other forms as well, warranting further research.

In parallel to the development of APS-2 our patient had a dramatic, sustained CR of her advanced melanoma after just 4 doses of pembrolizumab. She therefore demonstrated a response to PD1 inhibition that appeared unusually sensitive, both in terms of susceptibility to autoimmune toxicity and therapeutic efficacy. There is an ongoing effort to identify predictive biomarkers for response in patients treated with anti-PD1/PD-L1 agents. These primarily focus on characteristics displayed by the tumor such as the character and localisation of inflammatory cell infiltrates, immune checkpoint expression and gene expression in the tumor microenvironment and T cell markers [19–21]. Other host factors such as the content and diversity of fecal microbiome also appear to be important and have attracted considerable recent attention [22].

In contrast, relatively little is known about the predictive value of inherited host factors, with only one study (to our best knowledge) exploring the association between HLA haplotypes and treatment response. Through careful analysis of tissue from 1535 advanced cancer patients treated with ICIs, Chowell and colleagues noted significantly extended overall survival (OS) for patients with the HLA-B44 supertype and conversely worse survival with the HLA-B62 supertype. An exploratory analysis found a similar poor association with HLA-DP homozygosity, implying a potential role for HLA class II influencing patient’s response to these therapies [23].

As a clinical biomarker, there is also emerging evidence that the development of certain irAEs during anti-PD1/PD-L1 axis therapies is associated with treatment response. The most common endocrinological irAE is thyroiditis [1], associated with a significant progression-free and OS benefit in a retrospective series of NSCLC patients treated with such therapy [7]. Similarly, in another prospective cohort of NSCLC treated with PD1 therapy, those patients experiencing skin toxic effects also had improved OS and PFS. Strikingly, analysis of infiltrating T cells from matched tumor and skin biopsy samples revealed identical T-cell receptor sequences, indicating the same T cell clonotype reacting against shared antigens (in tumor and normal tissue). To our knowledge these are the first data to shed light on a mechanism for the association between irAE and anti-cancer response [24].

Unfortunately, our patient would now likely need lifelong hormone replacement with insulin, corticosteroids and thyroxine, having experienced considerable morbidity from her anti-cancer therapy. This case serves as a reminder to our group and all clinicians to vigilantly monitor for immune related endocrinopathies in patients receiving anti-PD1/PD-L1 inhibitors, even within the first cycle of therapy. As suggested by the Society for Immunotherapy of Cancer Toxicity Management Working Group a pre-treatment baseline TSH, free T4, 8 am ACTH, 8 am cortisol, glucose and HbA1c should be considered in all patients prior to immune checkpoint inhibitor therapy. Clinicians should then consider routine monitoring of patients’ early morning ACTH and cortisol (every month for 6 months, then every 3 months for 6 months, then every 6 months for 1 year) [25].

## Additional file

Additional file 1: Search strategy developed for the Ovid MEDLINE® database (DOCX 15 kb)

## Data Availability

Not applicable.

## Abbreviations

AJCC: American Joint Committee on Cancer
Anti-GAD: Anti-Glutamic Acid Decarboxylase
Anti-IA2: Anti-islet antibody 2
Anti-PD1: Anti-programmed cell death protein 1
APS-2: Autoimmune polyendocrine syndrome type II
CR: Complete response
CTLA-4: Cytotoxic T lymphocyte-associated antigen 4
DM: Diabetes mellitus
FDG: Fluorodeoxy-glucose
HLA: Human Leukocyte Antigen
ICI: Immune checkpoint inhibitor
irAEs: Immune related adverse events
MeSH: Medical Subject Headings
NSCLC: Non-small cell lung cancer
OS: Overall survival
PD1: Programmed cell death protein 1
PD-L1: Programmed death ligand 1
PET: Positron emission tomography
RCC: Renal cell carcinoma
T1DM: Type 1 diabetes mellitus
TSH: Thyroid stimulating hormone
