# Supplementary material for: Anti-programmed cell death protein 1 (anti-PD1) immunotherapy induced autoimmune polyendocrine syndrome type II (APS-2): a case report and review of the literature
Source: J Immunother Cancer. 2019 Sep 5;7:241. doi: 10.1186/s40425-019-0713-y (PMC6729071; doi:10.1186/s40425-019-0713-y)
Supplement: Supplementary file 1 — Search strategy developed for the Ovid MEDLINE® database (DOCX 15 kb) [file 40425_2019_713_MOESM1_ESM.docx]

Additional file 1

Search strategy developed for the Ovid MEDLINE® database

Run 29^th^ April 2019

|  |  |
| --- | --- |
| 1. endocrinopathy.mp. [mp=title, abstract, original title, name of substance word, subject heading word, floating sub-heading word, keyword heading word, organism supplementary concept word, protocol supplementary concept word, rare disease supplementary concept word, unique identifier, synonyms] |  |
| 2. autoimmune polyendocrine syndrome.mp. [mp=title, abstract, original title, name of substance word, subject heading word, floating sub-heading word, keyword heading word, organism supplementary concept word, protocol supplementary concept word, rare disease supplementary concept word, unique identifier, synonyms] |  |
| 3. autoimmune polyglandular syndrome.mp. [mp=title, abstract, original title, name of substance word, subject heading word, floating sub-heading word, keyword heading word, organism supplementary concept word, protocol supplementary concept word, rare disease supplementary concept word, unique identifier, synonyms] |  |
| 4. schmidt syndrome.mp. [mp=title, abstract, original title, name of substance word, subject heading word, floating sub-heading word, keyword heading word, organism supplementary concept word, protocol supplementary concept word, rare disease supplementary concept word, unique identifier, synonyms] |  |
| 5. Polyendocrinopathies, Autoimmune/ |  |
| 6. endocrinopathies.mp. [mp=title, abstract, original title, name of substance word, subject heading word, floating sub-heading word, keyword heading word, organism supplementary concept word, protocol supplementary concept word, rare disease supplementary concept word, unique identifier, synonyms] |  |
| 7. 1 or 2 or 3 or 4 or 5 or 6 |  |
| 8. Programmed Cell Death 1 Receptor/ |  |
| 9. Immunotherapy/ |  |
| 10. pembrolizumab.mp. [mp=title, abstract, original title, name of substance word, subject heading word, floating sub-heading word, keyword heading word, organism supplementary concept word, protocol supplementary concept word, rare disease supplementary concept word, unique identifier, synonyms] |  |
| 11. nivolumab.mp. [mp=title, abstract, original title, name of substance word, subject heading word, floating sub-heading word, keyword heading word, organism supplementary concept word, protocol supplementary concept word, rare disease supplementary concept word, unique identifier, synonyms] |  |
| 12. atezolizumab.mp. [mp=title, abstract, original title, name of substance word, subject heading word, floating sub-heading word, keyword heading word, organism supplementary concept word, protocol supplementary concept word, rare disease supplementary concept word, unique identifier, synonyms] |  |
| 13. avelumab.mp. [mp=title, abstract, original title, name of substance word, subject heading word, floating sub-heading word, keyword heading word, organism supplementary concept word, protocol supplementary concept word, rare disease supplementary concept word, unique identifier, synonyms] |  |
| 14. durvalumab.mp. [mp=title, abstract, original title, name of substance word, subject heading word, floating sub-heading word, keyword heading word, organism supplementary concept word, protocol supplementary concept word, rare disease supplementary concept word, unique identifier, synonyms] |  |
| 15. 8 or 9 or 10 or 11 or 12 or 13 or 14 |  |
| 16. 7 and 15 |  |
